# Comparison of insulin-like growth factor-1 and sclerostin levels between premenopausal women with and without diabetes mellitus

**DOI:** 10.1016/j.jtumed.2021.05.007

**Published:** 2021-06-11

**Authors:** Mahriani Sylvawani, Bambang Setyohadi, Dyah Purnamasari, Murdani Abdullah, Muhammed R. Kurniawan

**Affiliations:** a Division of Rheumatology, Department of Internal Medicine, School of Medicine, Universitas Syiah Kuala, Banda Aceh, Indonesia; b Division of Rheumatology, Department of Internal Medicine, Faculty of Medicine Universitas Indonesia, Cipto Mangunkusumo Hospital, Jakarta, Indonesia; c Division of Metabolism and Endocrinology, Department of Internal Medicine, Faculty of Medicine, Universitas Indonesia, Cipto Mangunkusumo Hospital, Jakarta, Indonesia; d Division of Gastroenterology, Department of Internal Medicine, Faculty of Medicine Universitas Indonesia, Cipto Mangunkusumo Hospital, Jakarta, Indonesia

**Keywords:** داء السكري, قبل انقطاع الطمث, عامل النمو الشبيه بالأنسولين -١, سكليروستين, بانيات العظم, Diabetes mellitus, IGF-1, Osteoblasts, Premenopause, Sclerostin

## Abstract

**Objectives:**

This study assesses the serum levels of insulin-like growth factor-1 (IGF-1) and sclerostin as markers of decreased bone formation in premenopausal women with type 2 diabetes mellitus.

**Methods:**

A cross-sectional study was conducted to measure serum levels of IGF-1 and sclerostin in 40 premenopausal women with and without diabetes mellitus using an enzyme-linked immunosorbent assay. The levels of IGF-1 and sclerostin were compared between the groups using the Mann Whitney test and unpaired t-test, respectively.

**Results:**

The median IGF-1 serum levels were 40.60 ng/mL and 42.7 ng/mL in the diabetic and non-diabetic groups, respectively, with no significant difference. The serum levels of sclerostin were significantly higher in the diabetic group than in the non-diabetic group (132.1 pg/mL and 96.0 pg/mL, respectively; *p* < 0.001).

**Conclusion:**

The levels of sclerostin were significantly higher in premenopausal women with diabetes mellitus than in the non-diabetic group. Since sclerostin influences the differentiation and maturation of osteoblasts, serum sclerostin might potentially be useful as a marker of decreased bone formation in premenopausal women with diabetes.

## **الملخص**

**أهداف البحث:**

تقيم هذه الدراسة مستويات المصل لعامل النمو الشبيه بالأنسولين -١ وسكلروستين كعلامات لانخفاض تكوين العظام لدى النساء قبل انقطاع الطمث المصابات بداء السكري من النوع ٢.

**طرق البحث:**

تم إجراء دراسة مقطعية حيث تم قياس مستويات مصل عامل النمو الشبيه بالأنسولين -١ وسكلروستين في مصل ٤٠ امرأة قبل انقطاع الطمث مع أو بدون داء السكري باستخدام مقايسة الممتز المناعي المرتبط بالإنزيم. وتمت مقارنة مستويات عامل النمو الشبيه بالأنسولين -١ وسكلروستين بين المجموعات.

**النتائج:**

كان متوسط مستويات عامل النمو الشبيه بالأنسولين -١ في الدم ٤٠.٦٠نانوغرام / مل و٤٢.٧ نانوغرام / مل في المجموعات المصابة بالسكري وغير المصابة بالسكري، على التوالي مع عدم وجود فرق معنوي. كانت مستويات سكليروستين في المصل أعلى بكثير في مجموعة مرضى السكري مقارنة بالمجموعة غير المصابة بالسكري، ١٣٢.١ بيكوغرام / مل و٩٦.٠بيكوغرام / مل، على التوالي.

**الاستنتاجات:**

كانت مستويات سكليروستين أعلى بشكل ملحوظ في النساء في فترة ما قبل انقطاع الطمث المصابات بداء السكري مقارنة مع غير المصابات بداء السكري. نظرا لأن سكلروستين يؤثر على تمايز ونضج بانيات العظم، فقد يكون من المحتمل أن يكون سكلروستين المصل مفيدا كعلامة على انخفاض تكوين العظام لدى النساء المصابات بالسكري قبل انقطاع الطمث.

## Introduction

Individuals with diabetes are at a higher risk of bone fractures than the general population.^1^ This risk is associated with the bone mass density (BMD) degradation and bone tissue changes that result in decreased bone strength and quality, known as diabetoporosis.^2^ There are some mechanisms associated with diabetoporosis. A high concentration of glucose is the main energy source for the osteoclast activity responsible for the dissolution and absorption of bones, and it plays an important role in the non-enzymatic glycosylation of several bone proteins, leading to decreased bone quality.^3^ Hyperglycaemia also decreases osteoblast gene expression and maturation, resulting in declining levels and functions of osteoblasts, which are the cells responsible for the synthesis and mineralisation of bones.^4^ Furthermore, the use of glitazone as an oral hypoglycaemic agent stimulates the nuclear receptor peroxisome proliferator-activated receptor gamma (PPAR-γ) and thus decreases bone density by inhibiting osteoblast activation and differentiation.^5^ Insulin, which has an osteogenic effect, directly increases the number and function of osteoblasts, and indirectly controls the levels of blood glucose, insulin-like growth factor-1 (IGF-1), parathyroid hormone, and vitamin D.^4^ Therefore, the lack of insulin in diabetic patients might decrease bone formation. However, a study has suggested that obesity and insulin resistance in diabetic patients can increase bone formation through a hyperinsulinaemia mechanism.^6^ Other conditions such as diabetic nephropathy and diabetic neuropathy, and a history of diabetes for more than 10 years are also associated with a higher risk of osteoporosis in patients with diabetes.^7^

Bone strength or resistance is determined by bone density and quality. Bone density can be measured by BMD examinations using dual-energy X-ray absorptiometry (DXA), whereas bone quality is determined by bone microstructure examination.^8^ Although BMD is widely used to screen and diagnose osteoporosis, a systematic review showed that BMD examination is not sensitive for diabetic patients as it was reduced in some studies, increased in others, or remained unchanged.^9^

Post-menopausal women are also at a higher risk of fracture. When this is due to a decrease in oestrogen, it is known as primary osteoporosis, and when it is due to certain drugs or underlying diseases such as diabetes, it is known as secondary osteoporosis and can occur in both pre- and post-menopausal women.^10^ Thus, this increases the risk of fracture in women with diabetes, and early assessment of bone fragility could be used to screen for fracture and osteoporosis risk among women with diabetes.

Although potential markers of osteoporosis in diabetic women have been previously proposed, here we propose IGF-1 and sclerostin as potential markers as well. IGF-1, a protein produced by the liver, has been suggested to increase osteoblast recruitment, bone matrix formation, and collagen synthesis.^11^, ^12^, ^13^ Insulin deficiency and hyperglycaemia, changes in advanced glycation end products (AGEs) axis, decreased IGF-1, and changes in osteocalcin are some factors that lead to decreased bone quality in diabetic patients.^14^ Hyperglycaemia inhibits the secretion of growth hormones, which eventually decreases IGF-1, leading to decreased osteoblast proliferation, collagen synthesis, and bone matrix formation, all of which further result in decreased bone quality.^13^ Sclerostin, a glycoprotein produced by osteocytes, inhibits the Wnt signalling pathway (a signalling pathway that affects bone modelling and remodelling) and leads to decreased bone formation.^14^ Increased serum sclerostin levels have been reported in both type 1 and type 2 diabetes patients.^15^^,^^16^ Hyperglycaemia in diabetic patients directly increases the production of sclerostin and therefore inhibits bone formation by downregulating the Wnt pathway. Moreover, hyperglycaemia indirectly accumulates AGEs in the bone collagen, leading to increased sclerostin.^14^ The stud studies also suggested that increased sclerostin was associated with an increased risk of spine fracture.^17^^,^^18^

We hypothesised that low IGF-1 and high sclerostin might act as markers for decreased bone formation in diabetic patients as they are associated with a higher risk of osteoporosis and fractures. However, most of the studies on bone metabolism in diabetic patients were conducted among men and post-menopausal women, which might have led to biased results due to hormonal effects. To our knowledge, there is no published study on the serum levels of IGF-1 and sclerostin as markers of decreased bone formation in premenopausal women with diabetes. This study was conducted to compare the levels of IGF-1 and sclerostin in premenopausal women with and without diabetes, as they might be useful as markers of decreased bone formation in premenopausal women with type 2 diabetes.

## Materials and Methods

### Study design and setting

This is a part of a previously published study^19^ that showed a decrease in bone formation and resorption in premenopausal women with diabetes, marked by low levels of procollagen type 1 amino-terminal propeptide (P1NP) and cross-linked telopeptide of type 1 collagen (CTX) as an early sign of diabetoporosis. Utilising a cross-sectional design, the study enrolled premenopausal women aged >35 years with at least a 5-year history of diabetes. Premenopausal women were chosen to avoid the bias caused by the natural osteoporosis process that occurs in postmenopausal women. The cut-off age was chosen as 35 years as it was above the age of peak bone mass formation and therefore, would not affect the bone turnover measurements and other bone metabolism markers.

### Participants

Forty premenopausal women aged >35 years who had been diagnosed with type 2 diabetes for at least 5 years were enrolled. The same number of non-diabetic female controls was selected by matching for age and body mass index (BMI) with the diabetic cases. Women with grades 4–5 of chronic kidney disease, hepatic diseases (e.g. hepatitis and cirrhosis), history of bone metabolism disorders (e.g. hyperparathyroidism, Paget's disease, osteomalacia, or osteogenesis imperfecta), thyroid disorders, glucocorticoid consumption for more than 3 months within the last 3 years, or consumption of drugs that could alter bone metabolism (e.g. bisphosphonate, hormonal therapy like oestrogen), and those who were first diagnosed with diabetes before 25 years of age, were excluded from the study. The participants were recruited from several hospitals and primary health centres (known locally as *Puskesmas*) in Jakarta in 2017 using a consecutive sampling technique. The number of samples recruited for this study was calculated based on previous studies about the levels of IGF-1 and sclerostin.^20^^,^^21^ The minimum sample to obtain 80% statistical power for the IGF-1 study was 31, whereas that for the sclerostin study was 6. We decided to recruit 40 samples to meet these minimum requirements.

### Measurements

The level of IGF-1 was measured from preserved serum by enzyme-linked immunosorbent assay (ELISA) using a Human IGF-I/IGF-1 Quantikine ELISA Kit (R&D Systems, Minneapolis, MN, USA), according to the manufacturer's protocol. The level of serum sclerostin was measured using Human Sclerostin Quantikine ELISA Kit (R&D Systems, Minneapolis, MN, US), as per the manufacturer's protocol. The assays have detection ranges of 0.1–6 ng/mL and 31.3-2,000 pg/mL, respectively.

Demographic information, clinical information, and essential laboratory parameters (age, occupation, body weight, diabetic treatment, HbA1C level, glomerular filtration rate, creatinine level, and alanine transaminase level) were also collected.

### Data analysis

The levels of IGF-1 and sclerostin were compared between the diabetic and non-diabetic groups using the Mann–Whitney test and unpaired t-test, based on assessment of the normality distribution of the data. Data analysis was conducted using SPSS ver.22.

## Results

A total of 80 participants were enrolled and analysed in this study. Of these, 40 were premenopausal diabetic women and 40 were premenopausal women without diabetes. The participant characteristics are presented in Table 1.

**Table 1:** Participants' characteristics.

Characteristics	Diabetic group (n = 40)	Non-diabetic group (n = 40)
Age (year), median (range)	45 (41–48)	39 (37–45)
Has had diabetes (year), median (range)	8 (5–11)	0 (0–0)
Occupation, n (%)		
Civil servants	12 (30.0)	20 (50.0)
Private sectors	6 (15.0)	14 (35.0)
House wives	22 (55.0)	6 (15.0)
BMI (kg/m^2^), mean	26.97	26.15
BMI Categories, n (%)		
Obese	25 (62.5)	22 (55.0)
Overweight	4 (10.0)	7 (17.5)
Normal	11 (27.5)	11 (27.5)
HbA1C level (%), mean	9.54	5.46
HbA1C classification, n (%)		
≤7%	7 (17.5)	40 (100.0)
>7%	33 (82.5)	0 (0.0)
eGFR (mL/min/1.73 m^2^), median (range)	107.6 (90.0–118.0)	110.7 (106.2–116)
Creatinine (mg/dL), median (range)	0.60 (0.50–0.80)	0.70 (0.60–0.21)
Alanine transaminase (U/L), median (range)	18.5 (11.0–24.7)	16.0 (11.0–54.0)
Diabetes treatment, n (%)		
Insulin	22 (55.0)	0 (0.0)
Oral anti-diabetic	18 (45.0)	0 (0.0)

BMI: bone mass density; eGFR: estimated glomerular filtration rate; HbA1C: glycated hemoglobin.

The median age of the diabetic group was 45 years (range, 41–48 years), whereas the median age of the control group was 39 years (range, 37–45 years). More than half (55%) the participants were housewives, and half (50%) of the women in the control group were civil servants. The mean BMIs were 26.97 kg/m^2^ and 26.15 kg/m^2^ in the diabetic and non-diabetic groups, respectively. More women in the diabetic group were obese compared to those in the non-diabetic group (62.5% *vs.* 55%); the percentages of overweight participants were 10.0% and 17.5% in the diabetic and non-diabetic groups, respectively.

Within the diabetic group, the median number of years of having diabetes was 8 years since being diagnosed with diabetes (range, 5–11 years); most of them (82.5%) had HbA1C of >7%, and more than half (55%) used insulin. The median estimated glomerular filtration rates (eGFR) were 107.6 mL/min/1.73 m^2^ and 110.7 mL/min/1.73 m^2^ in the diabetic and non-diabetic groups, respectively.

The median level of IGF-1 was 40.6 ng/mL in the diabetic group and 42.7 ng/mL in the non-diabetic group (Table 2). The analysis showed that the level of serum IGF-1 tended to be lower in diabetic patients than in non-diabetic participants, although the difference was not significant (p = 0.900).

**Table 2:** The serum level of IGF-1 and sclerostin in premenopausal women with and without diabetes.

Variable	Groups		p-value
	Diabetic (n = 40)	Non-diabetic (n = 40)	
IGF-1 serum (ng/mL), median (range)^a^	40.6 (11–110)	42.7 (10–65)	0.900
Sclerostin (pg/mL), mean^b^	132.1 (SD ± 41.54)	96.0 (SB±43.66)	<0.001

a Analyzed with Mann Whitney test.

b Analyzed with unpaired t-test.

Our study found that the mean level of serum sclerostin in the diabetic group was higher than that in control group, at 132.1 pg/mL and 96.0 pg/mL, respectively (Table 2). Unpaired t-test revealed statistical significance with p < 0.001.

## Discussion

Although BMD has been used to screen and diagnose osteoporosis, it is not sensitive for diabetic patients.^9^ We compared the levels of IGF-1 and sclerostin in premenopausal women with and without diabetes as potential markers of diabetoporosis in premenopausal women with diabetes. Our study found that the median level of IGF-1 in diabetic individuals was 40.6 ng/mL. This was lower than that found in a previous study of 83 participants in Egypt, which showed a median serum IGF-1 level of 158 ng/mL in both male and female diabetic patients.^22^ Although we found that the IGF-1 level in the diabetes group was lower than that in the non-diabetic group, this was not statistically significant. A previous study in 2016 found significantly lower IGF-1 levels in diabetic individuals compared to non-diabetic controls.^23^ These results might be due to differences in the characteristics of study participants, such as BMI, years of having diabetes, and therapeutic regimen used.

Our study found that the levels of serum sclerostin were significantly higher in diabetic participants than in the control group. This finding was in line with previous studies that suggested that levels of serum sclerostin increased in people with diabetes and were associated with spine fracture.^17^^,^^24^ A previous study also suggested that sclerostin levels were positively associated with the years of having diabetes and glycated haemoglobin levels, and inversely associated with bone turnover markers.^25^ In the present study, most of the participants had suffered from diabetes for more than 5 years, which might explain the high sclerostin serum levels. Hyperglycaemia in diabetic patients increases proinflammatory cytokines (interleukin-1 and interleukin-6), which induce osteocytes to produce sclerostin. Sclerostin hinders bone formation by inhibiting the Wnt signalling pathway.^14^ Hyperglycaemia also indirectly increases sclerostin levels by accumulating AGEs inside the bone collagen.^14^ A previous study also reported that sclerostin levels increased in type 2 diabetic patients, independent of age and sex, and were correlated with the duration of diabetes, glycated haemoglobin levels, and bone mass density.^24^ This explains why the sclerostin level in participants with diabetes in this study was higher than that in healthy controls.

There are some limitations to this study that need to be discussed. First, the number of participants in this study was relatively small; therefore, further studies with larger sample sizes are required. Second, the present study did not measure the BMD and hormonal status of the patients, limiting our assessment of the association between markers and bone mineralisation, and between hormonal status and bone metabolism. Nevertheless, this study provides evidence for the potential use of serum sclerostin as a marker of diabetoporosis in premenopausal women.

## Conclusion

We found no difference in IGF-1 levels between premenopausal women with and without diabetes. Our data indicate that premenopausal women with diabetes have significantly higher serum levels of sclerostin than the non-diabetic group. This suggests that the increased level of sclerostin might be used as a marker of decreased bone formation in premenopausal women with type 2 diabetes.

## Recommendations

Serum sclerostin might potentially be useful as a marker of decreased bone formation in premenopausal women with diabetes and therefore needs to be measured to prevent diabetoporosis in premenopausal women.

## Source of funding

This research did not receive any specific grant from funding agencies in the public, commercial, or not-for-profit sectors.

## Conflict of interest

The authors have no conflict of interest to declare.

## Ethical approval

The study protocol was approved by the Ethical Committee for Health Research, School of Medicine, Universitas Indonesia (090/UN2.F1/ETIK/2017). A research permit from the Research Unit, Cipto Mangunkusumo Hospital, with number 1380a/UN2.F1/ETIK/XII/2018, was obtained prior to the study (1 July 2018). All participants provided written informed consent prior to participating in the study.

## Authors' contribution

MS was responsible for conceptualisation of the study, validation of the data, analysis of the data, data curation, original draft preparation, and reviewing the manuscript. BS was responsible for conceptualisation of the study, validation of the data, review of the manuscript, and supervision of the study. DP conceived and designed the study, took part in the validation of the data, and supervised the study. MA validated the data and supervised the study. MRK validated the study and wrote the manuscript. All authors critically reviewed and approved the final draft and are responsible for the content and similarity index of the manuscript.

## References

[1] Yamamoto M., Yamaguchi T., Yamauchi M., Kaji H., Sugimoto T. (2009). Diabetic patients have an increased risk of vertebral fractures independent of BMD or diabetic complications. J Bone Miner Res.

[2] Board HRaD (2013).

[3] Bridges M., Moochhala S., Barbour J., Kelly C. (2005). Influence of diabetes on peripheral bone mineral density in men: a controlled study. Acta Diabetol.

[4] Ghodsi M., Larijani B. (2016). Mechanisms involved in altered bone metabolism in diabetes: a narrative review. J Diabetes Metab Disord.

[5] Lecka-Czernik B. (2010). PPARγ, an essential regulator of bone mass: metabolic and molecular cues. IBMS BoneKEy.

[6] Milczarczyk A. (2008). Osteoporosis and bone fractures in patients with diabetes mellitus. Diabetol Do´swiadczalna i Klin.

[7] Baim S., Miller P. (2009). Perspective assessing the clinical utility of serum CTX in postmenopausal osteoporosis and its use in predicting risk of osteonecrosis of the jaw. J BONE Miner Res J Bone Min Res.

[8] Licata A. (2009). Bone density vs bone quality: what's a clinician to do. Cleve Clin J Med.

[9] Janghorbani M., Dam R.V., Willett W., Hu F. (2007). Systematic review of type 1 and type 2 diabetes mellitus and risk of fracture. Am J Epidemiol.

[10] Mirza F., Canalis E. (2015). Secondary osteoporosis: pathology and management. Eur J Endocrinol.

[11] Ferrari S., Abrahamsen B., Napoli N., Akesson K., Chandran M., Eastell R. (2018). Diagnosis and management of bonde fragility in diabetes: an emerging challange. Osteoporos Int.

[12] Jiang N., Xia W. (2018). Assessment of bone quality in patients with diabetes mellitus. Osteoporos Int.

[13] Aguirre G., Ita J.D., Rdl Garza, Castilla-Cortazar I. (2016). Insulin-like growth factor-1 deficiency and metabolic syndrome. J Transl Med.

[14] Dumic-cule I., Ivanac G., Lucijanic T., Katicic D., Jurin I., Birkic D. (2018). Type 2 diabetes and osteoporosis : current knowledge. Endocrine Oncol Metabol.

[15] Gaudio A., Privitera F., Battaglia K., Torrisi V., Sidoti M.H., Pulvirenti I. (2012). Sclerostin levels associated with inhibition of the Wnt/β-catenin signaling and reduced bone turnover in type 2 diabetes mellitus. J Clin Endocrinol Metabol.

[16] Gennari L., Merlotti D., Valenti R., Ceccarelli E., Ruvio M., Pietrini M.G. (2012). Circulating sclerostin levels and bone turnover in type 1 and type 2 diabetes. J Clin Endocrinol Metabol.

[17] Yamamoto M., Yamauchi M., Sugimoto T. (2013). Elevated sclerostin levels are associated with vertebral fractures in patients with type 2 diabetes mellitus. J Clin Endocrinol Metab.

[18] Pacicca D.M., Brown T., Watkins D., Kover K., Yan Y., Prideaux M. (2019). Elevated glucose acts directly on osteocytes to increase sclerostin expression in diabetes. Sci Rep.

[19] Purnamasari D., Melisa P., Setyohadi B., Isbagio H. (2017). Low bone turnover in premenopausal women with type 2 diabetes mellitus as an early process of diabetes-associated bone alterations: a cross-sectional study. BMC Endocr Disord.

[20] Fang Y., Wang Z.Y., Mao Y., Xin H.T., Ren G.L., Bai X.F. (2006). Effects of insulin-like growth factor I on the development of osteoblasts in hyperglycemia. Diabetes Res Clin Pract.

[21] Xiaohong S., Difei W. (2017). Exploration of serum levels of SOST and its clinical significance in elderly patients with type 2 diabetes mellitus complicated with osteoporosis. Biomed Res.

[22] Amer N., Shaat A., Abo-EL-Aenin N., Abdel-Rasek A., Abdel-Gwad S. (2015). Osteoporosis in diabetics and its relation to IGF-1. Zagazig Univ Med J.

[23] Yamamoto M., Sugimoto T. (2016). Advanced glycation end products, diabetes, and bone strength. Curr Osteoporos Rep.

[24] García-Martín A., Rozas-Moreno P., Reyes-García R., Morales-Santana S., García-Fontana B., García-Salcedo J. (2012). Circulating levels of sclerostin are increased in patients with type 2 diabetes mellitus. J Clin Endocrinol Metab.

[25] Sanches C., Vianna A., Barreto F. (2017). The impact of type 2 diabetes on bone metabolism. Diabetol Metab Syndrome.

